# Metabolic Maturation Exaggerates Abnormal Calcium Handling in a *Lamp2* Knockout Human Pluripotent Stem Cell-Derived Cardiomyocyte Model of Danon Disease

**DOI:** 10.3390/biom13010069

**Published:** 2022-12-29

**Authors:** Robert J. Barndt, Qing Liu, Ying Tang, Michael P. Haugh, Jeffery Cui, Stephen Y. Chan, Haodi Wu

**Affiliations:** 1 Pittsburgh Heart, Lung, and Blood Vascular Medicine Institute, University of Pittsburgh School of Medicine, Pittsburgh, PA 15261, USA; 2 Department of Biological Sciences, Clemson University, Clemson, SC 29634, USA; 3 Center for Human Genetics, Clemson University, Greenwood, SC 29646, USA; 4 Department of Bioengineering, University of Pittsburgh Swanson School of Engineering, Pittsburgh, PA 15213, USA; 5 Department of Biological Sciences, University of Pittsburgh, Pittsburgh, PA 15260, USA; 6 Division of Cardiology, Department of Medicine, University of Pittsburgh School of Medicine, Pittsburgh, PA 15261, USA

**Keywords:** Danon disease, LAMP2, iPSC-derived cardiomyocyte, metabolic maturation, arrhythmia

## Abstract

Danon disease (DD) is caused by mutations of the gene encoding lysosomal-associated membrane protein type 2 (*LAMP2*), which lead to impaired autophagy, glycogen accumulation, and cardiac hypertrophy. However, it is not well understood why a large portion of DD patients develop arrhythmia and sudden cardiac death. In the current study, we generated *LAMP2* knockout (KO) human iPSC-derived cardiomyocytes (CM), which mimic the LAMP2 dysfunction in DD heart. Morphologic analysis demonstrated the sarcomere disarrangement in *LAMP2* KO CMs. In functional studies, *LAMP2* KO CMs showed near-normal calcium handling at base level. However, treatment of pro-maturation medium (MM) exaggerated the disease phenotype in the KO cells as they exhibited impaired calcium recycling and increased irregular beating events, which recapitulates the pro-arrhythmia phenotypes of DD patients. Further mechanistic study confirmed that MM treatment significantly enhanced the autophagic stress in the *LAMP2* KO CMs, which was accompanied by an increase of both cellular and mitochondrial reactive oxygen species (ROS) levels. Excess ROS accumulation in *LAMP2* KO CMs resulted in the over-activation of calcium/calmodulin dependent protein kinase IIδ (CaMKIIδ) and arrhythmogenesis, which was partially rescued by the treatment of ROS scavenger. In summary, our study has revealed ROS induced CaMKIIδ overactivation as a key mechanism that promotes cardiac arrhythmia in DD patients.

## 1. Introduction

Danon disease (DD) is clinically characterized by cardiomyopathy, skeletal myopathy, mental retardation, and visual problems [1]. As a rare multisystem disease, DD was first reported in 1981 by Danon and his colleagues as a glycogen storage disease, because the analysis of patient muscle biopsy identified excess glycogen particle accumulation in lysosomal vacuoles [2]. As biomedical research advances, we now know that DD is primarily caused by mutations in the lysosome-associated membrane protein (*LAMP2*) gene, which lead to the deficiency or absence of LAMP2 protein [3]. LAMP2 is a lysosomal membrane protein that plays a key role in the maturation of lysosomes and their fusion with autophagic vacuoles [4,5]. Thus, the deficiency of LAMP2 leads to accumulation of autophagic vacuoles in the cardiac and skeletal tissues of DD patients [6]. As an X-linked genetic disease, DD is more likely to affect male patients, who typically show early on-set hypertrophic cardiomyopathy (HCM) at age of 12 and eventually develop severe heart failure and arrhythmia around 20 years old [7,8]. Females with heterozygous *LAMP2* mutations can also be affected by DD with much milder symptoms [9]. To date, an increasing number of *LAMP2* mutations have been reported, yet the molecular mechanism of cardiac dysfunction and arrhythmia in DD patients remains unclear [10]. As a result, no specific therapy is currently available for DD patients [11]. 

To better understand the mechanism of DD, different animal models with LAMP2 deficiency have been created [3]. The first *LAMP2* KO mice model was generated in 2000, yet hemizygous male mice hardly survive longer than 40 days due to hemorrhagic infarction in different organs [12]. Similarly, other DD animal models, such as TALEN-generated hemizygous male rat and *LAMP2* knockout zebrafish, all exhibit an increased mortality rate at earlier ages [13,14]. Although typical autophagic vacuoles accumulation can be observed in the cardiac muscle of these DD models, most of them showed a much milder, if not different, cardiac phenotype compared to DD patients [15]. The significant phenotypic discrepancies between animal models and humans have greatly hampered the understanding of the molecular pathogenic mechanism of DD. Moreover, due to the rarity of DD and ethical concerns, it’s also challenging to conduct mechanistic studies with patient cardiac tissue. The combination of human induced pluripotent stem cells (iPSCs) platform and CRISPR genome-editing tools have provided great platforms for the in vitro study of DD. A couple of reports have described the modeling of cardiomyopathy using DD patient-specific iPSC-derived cardiomyocytes (iPSC-CMs) [16,17,18,19]. While most of these models recapitulated the disrupted mitophagy flux in DD patients, iPSC-CMs from different studies/patients showed distinct signaling abnormalities, which may partially be due to the immaturity of the iPSC-CMs and the significant variations in their genetic backgrounds [20,21]. More recently, multiple approaches have been developed to further promote the maturity of iPSC-CMs after differentiation, which will unleash the full potential of iPSC-CMs in modeling and mechanistic studies of DD [22,23,24]. 

To better understand the molecular mechanisms of DD, we have generated isogenic *LAMP2* knockout (KO) iPSCs as a disease model using the CRISPR/Cas9 tool in the current study. Both control (Ctrl) and *LAMP2* KO iPSCs were differentiated into iPSC-CMs, which were treated with maturation medium (MM) to promote metabolic and functional maturity [22]. The MM-treated *LAMP2* KO iPSC-CMs was able to recapitulate impaired autophagic flux, sarcomere disarrangement, and pro-arrhythmic calcium handling in DD cardiomyocytes. Our study further indicates deficiency of mitochondria results in ROS overload and CaMKII activation, which likely leads to pro-arrhythmic calcium handling in DD cardiomyocytes. The current study also showed that repressing ROS levels with a ROS scavenger compound can partially restore calcium homeostasis in our *LAMP2* KO iPSC-CM model. In summary, our findings help fill the gap in our understanding of the cellular mechanisms underlying DD heart arrhythmogenesis and have revealed novel therapeutic targets. 

## 2. Materials and Methods

### 2.1. Maintenance and Differentiation of Human iPSCs

The parental iPSC lines were acquired from Stanford University Cardiovascular Institute (SCVI) BioBank (https://med.stanford.edu/scvibiobank.html, MTA#00007023). The iPSC lines were maintained in Essential 8 (E8) Basal medium (Thermo Fisher Scientific, Waltham, MA, USA) on Matrigel-coated plates (Corning, NY, USA) at 37 °C with 5% oxygen and 5% CO_2_ without antibiotics. Cells were dissociated using DPBS (Life Technologies) supplemented with 0.5 µM EDTA (Sigma, St. Louis, MO, USA) and plated in medium with 10 µM Y-27632 dihydrochloride (Med Chem Express, Monmouth Junction, NJ, USA). After 24 h, medium without Y-27632 was used and changed every 24–48 h until passage. 

For cardiomyocyte differentiation, iPSCs were cultured to confluency and then differentiated with sequential medium changes as previously described [25]. Briefly, iPSCs were rinsed with RPMI medium supplemented with B27 minus insulin (Thermo Scientific) and then exposed to 6 µM CHIR99021 (Selleck Chem, Houston, TX, USA) for 48 h. Next, cells were switched back to RPMI with B27 minus insulin for 24 h and then changed to the same medium with 5 µM IWR (Selleck Chem) for 48 h. Afterward, the medium was changed to RPMI with B27 minus insulin for 48 h and finally RPMI with regular B27 for 48 h. This medium was used for the regular maintenance of iPSC-CMs for subsequent steps and experiments. iPSC-CMs were passaged using 10× TrypLE (Life Technologies, Carlsbad, CA, USA). For germ layer differentiation, iPSCs were plated onto Matrigel-coated Lab-Tek II glass chamber slides (Nunc, Rochester, NY, USA) at variable densities in E8 medium. After 48 h, medium was replaced and changed every day with STEMdiff Trilineage medium (StemCell Technologies, Vancouver, Canada) specific to each germ layer for 7 days. 

### 2.2. Metabolic Maturation of iPSC-CMs In Vitro

A maturation medium (MM) was prepared as previously described [22]. RPMI -glucose medium was supplemented with 3 mM D-(+)-glucose (Sigma), 10 mM sodium L-lactate (Sigma), 5 mM creatine monohydrate (Sigma), 2 mM taurine (Sigma), 2 mM L-carnitine hydrochloride (Sigma), 500 mM ascorbic acid 2-phosphate sesquimagnesium salt hydrate (Sigma), 1× MEM non-essential amino acid solution (Gibco, New York, NY, USA), 5 mg/ml vitamin B12 (Sigma), 0.82 mM biotin (Sigma), 0.5% AlbuMAX I lipid-rich BSA (Gibco), and 1% KnockOut Serum replacement (Gibco), then pH adjusted to 7.4 and 0.2 µm-filter sterilized (Millipore, Burlington, MA, USA). In the current study, iPSC-CMs were shifted to MM for 48 h prior to analysis.

### 2.3. Knockout of LAMP2 Using CRISPR/Cas9

**Figure 1:**
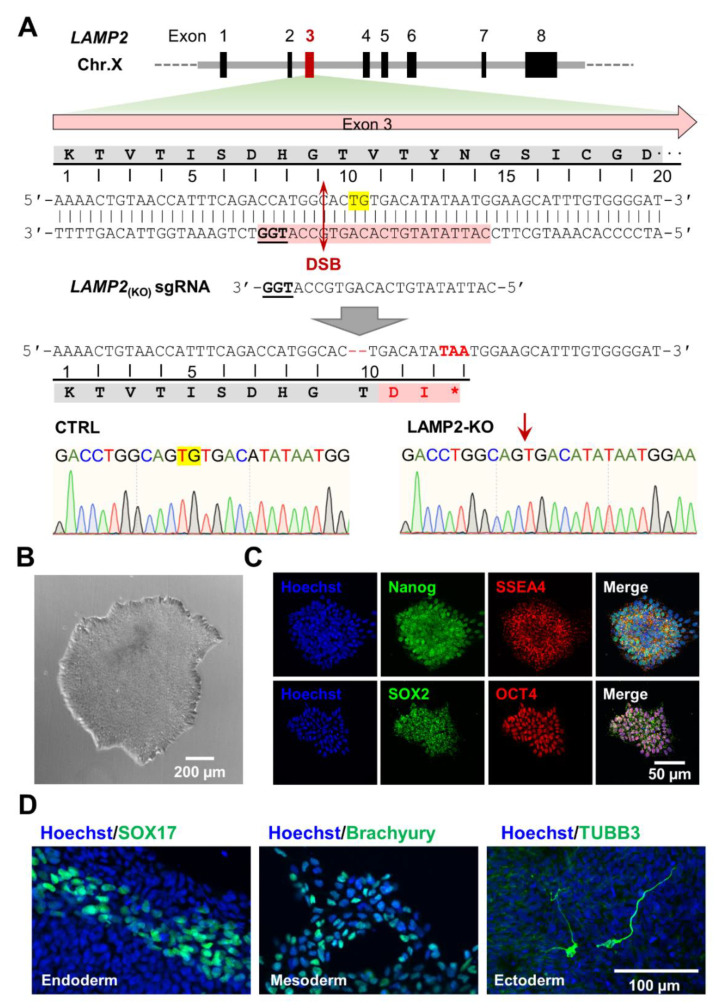
Generation of *LAMP2* knock out iPSC model of Danon disease. (**A**) Top: *LAMP2* gene structure and design of small guide RNA for CRISPR/Cas9 knockout. Middle: error-prone repairs of double strand break (DSB) induced a 2-base deletion in exon 3 of *LAMP2*, which results in an early stop codon that truncates LAMP2. Bottom: Sequence chromatograms showed the result of gene editing. Yellow background highlights the deleted nucleotides. * indicates stop codon that induce truncation of LAMP2; (**B**) *LAMP2* KO iPSCs exhibit the typical stem cell clone morphology as shown in phase contrast view; (**C**) Immuno-fluorescence staining shows the expression of pluripotency markers (Nanog, SSEA-4, SOX2, and OCT4) in *LAMP2* KO iPSCs; (**D**) In vitro 3-germ layer differentiation of *LAMP2* KO iPSCs was confirmed by the immunofluorescence staining of specific markers: SOX17 for endoderm, Brachyury for mesoderm, and γ-tubulin for ectoderm.

A small-guide RNA (sgRNA) targeting exon 3 of the *LAMP2* gene was designed using the online CRISPR design tool Benchling (https://benchling.com, access on 1 March 2022) (Figure 1A), and the BpiI compatible ends were then added to the cloning oligos. Forward CACCGCATTATATGTCACAGTGCCA and reverse AAACTGGCACTGTGACATATAATGC (IDT, NJ, USA) oligos were annealed and phosphorylated with T4 PNK (NEB, MA, USA), and then subcloned into FastDigest BpiI (Thermo Scientific) -treated pSpCas9-2A-Puro V2.0/PX459 (Addgene, MA, USA. Cat#62988) with T7 DNA ligase (NEB). The presence of the sgRNA was confirmed by Sanger sequencing using the U6 primer, GACTATCATATGCTTACCGT (IDT). 

For gene targeting, PX459-*LAMP2* KO sgRNA vector was delivered into control iPSCs through electroporation using a Nucleofector 2b device (Lonza, Basel, Switzerland) and Amaxa human stem cell (hSC) nucleofector kit 1 (Lonza). Briefly, sub-confluent iPSCs were pretreated with 10 µM Y-27632 for 1 h, dissociated using gentle cell dissociation reagent (StemCell Technologies), and resuspended in prewarmed DMEM/F12 medium. After cell counting, 8 × 10^5^ cells were mixed with 2 or 4 µg of vector in 100 µL of hSC nucleofector solution 1: supplement 1 at a ratio of 4.5:1, and then electroporated using program A-023. Afterward, cells were chased with E8 medium supplemented with Y-27632 and transferred to a Matrigel-pretreated 12-well plate. Y-27632 was removed from the medium the next day, and then attached cells were selected with 0.1 ug/ml puromycin for 24–36 h. Surviving clones were isolated and genotyped for the presence of indels as described [26]. Briefly, genomic DNA was isolated using QuickExtract DNA Extraction Solution (Lucigen, Middleton, WI, USA) according to the manufacturer’s instructions. The sgRNA-targeted *LAMP2* site was amplified using Q5 Hot Start High-Fidelity 2× Master Mix (NEB) and forward GCAGAAGAATCAGGGACTGG and reverse TCACTCAAGCAATGACAGCA primers at the final concentration of 0.25 µM. The following thermocycling program was used: 98 °C initial denaturation, 35 cycles 98 °C 10 s/60 °C 15 s/72 °C 20 s, then a final 72 °C 5 min, in a C1000 Touch Thermocycler (Bio-Rad, CA, USA). PCRs were run through a GeneJET (Thermo Scientific) column prior to Sanger sequencing (Azenta, MA, USA). For the generation of the second *LAMP2* KO iPSC line, another sgRNA was designed to target the exon 2 of the *LAMP2* (Supplemental Figure S1) and the following cloning oligos and designs were used: Forward strand cloning oligo: CACCGCATAAGACCGCACAGCTCC and reverse strand oligo: AAACGGAGCTGTGCGGTCTTATGC (IDT). For genotyping of the targeting region by this sgRNA, forward GTCACCAGTCTGAGCCATGA and reverse AAAAATCCCAGAGTTTCAGCA primers were used.

### 2.4. RNA Extraction of mRNA Expression Quantification by qPCR

Isolation of total RNA from iPSCs and iPSC-derived cardiomyocytes was performed using TRIzol (Ambion Life Technologies, Austin, TX, USA) according to manufacturer’s instructions. RNA was DNase-treated for 30 min then purified using Direct-zol RNA MicroPrep columns (Zymo Research, Irvine, CA, USA). Concentrations were determined using a NanoDrop 8000 spectrophotometer (Thermo Scientific). For reverse-transcription, iScript reverse transcription supermix for RT-qPCR (Bio-Rad) was used according to manufacturer’s instructions. The abundance of gene cDNAs was analyzed in triplicate using the QuantStudio5 analyzer (Applied Biosystems, Waltham, MA, USA) with QuantStudio design & analysis v1.5.2 software (Applied Biosystems) and iTaq universal SYBR green supermix (Bio-Rad) with 0.3 µM of each primer. Fast ramp speed and the following program were selected: 95 °C 20 s denaturation stage, then 40 cycles of 95 °C 1 s and 60 °C 20 s holding stage, followed by a melting curve stage. Relative gene expression was determined using the 2^-ΔΔCT^ method. The RT-qPCR primers used in the current study are listed in Supplemental Table S1. 

### 2.5. Protein Extraction and Western Blot Analysis

Whole cell protein lysates of iPSCs and iPSC-CMs were extracted using Pierce RIPA buffer (Thermo Scientific) supplemented with complete ULTRA EDTA-free protease inhibitor cocktail (Roche, Basel, Switzerland) and HALT protease & phosphatase inhibitor cocktail (Thermo Scientific). Total protein concentrations were determined using the Pierce BCA Protein Assay (Thermo Scientific) on a Synergy HT BioTek microplate reader at 562 nm and Gen5 v2.04 software (BioTek corporation, Winooski, VT, USA) with the standard curve method. 5 or 10 ug of total protein with 5× loading buffer (5% SDS, 50% glycerol, 250 mM Tris-HCl pH 6.8, 0.2% bromophenol blue, 15% β-mercaptoethanol (Sigma) was boiled for 10 min prior to loading on a 4–15% Mini-PROTEAN TGX stain-free gel (Bio-Rad) for 60 min at 125 V. WesternC precision plus protein standards (Bio-Rad) were run in parallel with samples to determine the protein molecular weight of individual bands. Gels were activated and imaged on a ChemiDoc XR system and Image Lab software (Bio-Rad) prior to and after transfer to PVDF membranes to ensure equal loading and transfer. Proteins were transferred using a Trans-blot Turbo (Bio-Rad) for 7 min at 1.3 A, 25 V. Membranes were placed in blocking solution (5% milk in TBS with 0.1% Tween 20, pH 7.6) for 1 h, then incubated overnight at 4 °C with primary antibody in blocking solution. After three TBST washes, membranes were treated with secondary horse anti-mouse IgG at 1:10,000 or goat anti-rabbit at 1:2500 HRP-linked antibody (Cell Signaling) in blocking solution. Following three TBST washes, bands were visualized using chemiluminescent Clarity Max Western ECL Substrate (Bio-Rad). The Western blot images were analyzed using an open-source software Fiji ImageJ (https://imagej.net/software/fiji/, accessed on 1 July 2022). The antibodies used in the current study are listed in Supplemental Table S2. 

### 2.6. Immunofluorescence Labeling of Fixed Samples

iPSCs and CMs were plated and grown on Lab-Tek II chamber slides pre-treated with Matrigel (1:250) in DMEM/F12 with L-glutamine, 15 mM HEPES (Sigma). Slides were fixed with 4% paraformaldehyde (Sigma) in 1× Phosphate Buffered Saline for 15 min, then washed in 1× PBS two times. Cells were then permeabilized for 1 h in 0.2% Triton X-100 (Acros Organics, Geel, Belgium) in PBS and blocked for 2 h with 5% bovine serum albumin (BSA) in PBS 0.2% Triton X-100. After two PBS washes, the slides were incubated overnight at 4 °C with primary antibody diluted in 1% BSA/0.1% Triton X-100 in PBS. After two 0.2% Tween 20 in 1× PBS and two 1× PBS washes, AlexaFluor secondary antibodies at 1:1000 dilution in 0.1% Triton X-100/1% BSA were incubated at room temperature (RT) in the dark for 1.5 h. Washes were repeated as before, then slides were stained with Hoechst 33,342 trihydrochloride trihydrate (Invitrogen, Waltham, MA, USA) at 1:5000 dilution in 1× PBS for 5 min at RT, followed by 1× PBS washes. Chambers were removed and ProLong Diamond Antifade mountant (Invitrogen) was added prior to sealing with fingernail polish. The antibodies used in the current study are listed in Supplemental Table S2.

### 2.7. Measurement of Cytosol and Mitochondria Reactive Oxygen Species (ROS)

To measure cytosolic ROS in live iPSC-CMs, cells were loaded with 5 µM CellROX™ Deep Red Reagent (Molecular Probes, OR, USA) and 2 µM Hoechst 33,342 (Invitrogen) at 37 °C for 30 min, and then washed 3 times with PBS. For mitochondrial morphology and ROS detection, iPSC-CMs were loaded with MitoTracker^®^ Green FM (Molecular Probes) at 200 nM and MitoSOX™ Red (Molecular Probes) at 5 µM for 15 min at 37 °C, then washed gently 3 times with warm buffer before imaging. For the treatment of maturation medium (MM), iPSC-CMs were switched to MM for 48 h before functional analysis. For the treatment of compounds, *N*-Acetylcysteine amide (NACA) was used at 5 mM and rapamycin used at 0.5 µM final concentration, respectively. For live cell imaging analysis, all the cells were seeded on Lab-Tek^®^ II chamber glass slides (Nunc) or glass-bottom 35 mm dishes with 14 mm microwell and No. 1.5 cover glass (MatTek, Ashland, MA, USA). 

### 2.8. Confocal Imaging and Image Analysis

Immunofluorescence slides and labelled live cell samples were imaged with a Nikon 1A confocal microscope, using either Nikon Plan Apo 20×/0.75 DIC, Nikon Plan Fluor 40× oil, 1.30NA, or Nikon Plan Apo OIL 60× oil, 1.40NA objectives (Nikon, Tokyo, Japan). Live cell indicators were imaged using the following combinations of excitation/emission setups: Hoechst 33,342 (350 nm/461 nm), CellROX™ Deep Red (633 nm/667 nm), MitoTracker™ Green FM (488 nm/515 nm), MitoSOX™ Red (520 nm/561 nm). For data analysis, the sarcomere arrangement signals were pulled out from each image and then further analyzed with Fast Flourier Transformation (FFT) in a customized IDL algorithm. Briefly, the digital immunostaining signals of α-actinin and TNNT2 will be transformed from a length domain into a power distribution in a frequency domain (Supplemental Figure S2D), and the main peak of the power distribution indicates the main period for the α-actinin and TNNT2 signals along the sarcomere. Higher power represents better regularity in signal distribution and more organized sarcomere structures. A more specific description can be found in the study by Wei et al. in 2010 [27]. Mitochondrial morphologies were analyzed using a MiNA plugin in Fiji ImageJ software according to a previous report [28]. Mitochondrial ROX level is presented as the value of the MitoSOX™ Red signal intensity normalized by the MitoTracker™ Green FM signal intensity. Cytosol ROX level is presented as the relative fluorescent unit (RFU) of CellROX™ Deep Red signal. 

### 2.9. Calcium Imaging and Data Analysis

For the analysis of spontaneous calcium activities in control and *LAMP2* KO cells, iPSC-CMs from both groups were seeded on Matrigel (Corning) pre-coated Lab-Tek^®^ II chamber slides and allowed to recover for 3–4 days until observed normal beating. Cells were loaded with 5 µM of Fluo-4 AM (Molecular Probe) for 10 min at 37 °C and then washed 3 times. The Fluo-4 AM signals of beating cells were recorded using a Nikon Ti-2E microscope platform (Nikon) customized for a high-content and high-frame-rate live cell functional imaging system with SPECTRA III Light Engine solid-state LED light source (Lumencor, Beaverton, OR, USA) and Hamamatus Orca-fusion Gen-III sCMOS camera (Hamamatus, Shizuoka, Japan). The videos of calcium dynamics were captured at 2048 × 2048 resolution and 50 fps frame rate. For ratiometric calcium imaging with Fura-2 AM, the iPSC-CMs from both groups were seeded in the center of 22 mm Matrigel pre-coated coverslips. After recovery, the cells were loaded with 5 µM Fura-2 AM with 0.1% F-127 for 10 min at RT in Tyrode’s solution (140 mM NaCl, 1 mM MgCl_2_, 5.4 mM KCl, 1.8 mM CaCl_2_, 10 mM glucose, and 10 mM HEPES, pH 7.4). Coverslips were then mounted on the imaging platform and paced at 0.5 HZ (10 volts/cm, bipolar pulse with 10 ms wave width) in a slotted bath imaging chamber with field stimulation (Warner Instrument LLC, Hamden, CT, USA, Cat#RC-21BRFS). The Fura-2 AM signals were captured as ratio pairs using 340/380 nm excitation and 510 nm emission at 2048 × 2048 resolution and 50 frames per ratio pairs rate. The cytosolic calcium signals in single cells were pulled out using Nikon NIS software, and then further analyzed with customized MATLAB (MathWorks, Natick, MA, USA) algorithms [29,30]. 

### 2.10. Plots and Statistical Analysis

For statistical analysis, unpaired t-test was used to compare two normally distributed data sets. One-way or two-way ANOVA followed by appropriate after-test methods were used in all pairwise comparisons among single or multiple groups of data. *p* < 0.05 was statistically significant. All data in bar plots were shown as mean ± SEM. For box plots, the error bar indicates the data distribution from 0–100%, the upper and lower edge of box indicate the value of data set at 75% and 25%, and the middle line indicates the median value of the data set. For scatter plots, the red line indicates the mean of the data set.

## 3. Results

### 3.1. Generation of Hemizygous LAMP2^y/-^ Knockout Human iPSCs

To generate a human *LAMP2* knockout iPSC line for the modeling of DD, *LAMP2* (KO) small guide RNAs (sgRNA) were designed using on-line CRISPR design tools at benchling.com to target the 3rd exon of *LAMP2* located on the X chromosome. (Figure 1A) The sgRNA oligos were cloned into the px458 gene-editing plasmid. The plasmid containing *LAMP2* (KO) sgRNA and the Cas9 was delivered into control male iPSCs using nucleofection [26]. After puromycin selection of transfected cells, the iPSCs were reseeded sparsely for single clone picking and genotyping. One of the iPSC clones was identified with a random dinucleotide deletion of “TG” in exon 3 which led to a frameshift in the open reading frame of LAMP2, and results in a truncated LAMP2 protein (Figure 1A). The clone was then expanded and characterized. Our results show the *LAMP2* KO iPSCs exhibit typical stem cell morphology (Figure 1B) and expresses pluripotency markers such as Nanog, SSEA4, Sox2, and Oct4 (Figure 1C). The pluripotency of *LAMP2* KO iPSCs was further confirmed with in vitro 3-germ layer differentiation assays, as evidenced by the positive markers of endoderm (SOX17), mesoderm (Brachyury), and ectoderm (γ-tubulin) (Figure 1D). Using the same method, we were able to generate a second *LAMP2* KO line by targeting a different locus in the exon 2 of *LAMP2*. Detailed sgRNA design and genome editing results are presented in supplemental figures (Supplemental Figure S1). To confirm the specificity of our sgRNA and the integrity of genomic sequence of the isogenic iPSC lines generated, the top 5 predicted off-target genomic sites of each sgRNA were genotyped using specifically designed primers sets (Supplemental Table S3), and our result show no notable genetic mutations in these genomic regions. 

### 3.2. LAMP2 Knockout Led to Morphological Remodeling of iPSC-Derived Cardiomyocytes 

**Figure 2:**
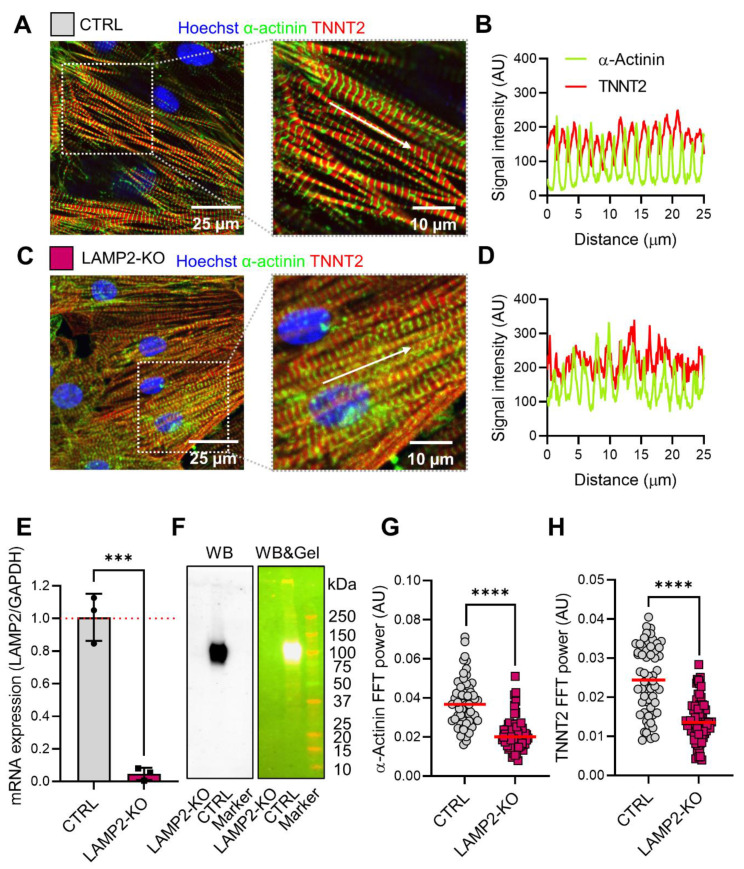
Morphological changes in *LAMP2* KO iPSC-CMs. (**A**) Immunofluorescence staining of Ctrl iPSC-CMs with sarcomere markers (green for α-actinin and red for TNTN2) with detailed sarcomere structures shown in zoom-in view. White arrows indicate the sarcomere arrangement analyzed in detail in panel B; (**B**) Distribution pattern of α-actinin and TNNT2 signals along the sarcomere; (**C**,**D**) Immunofluorescence staining of sarcomere proteins in *LAMP2* KO iPSC-CMs showed an irregular distribution of actinin and TNNT2 signals; € Quantification of mRNA expression of LAMP2 in both Ctrl and *LAMP2* KO iPSC-CMs. Results from 3 independent experiments for Ctrl and *LAMP2* KO group. Red dashed line indicates the ratio of LAMP2/GAPDH in Ctrl group was normalized to 1; (**F**) Western blot confirms the absence of LAMP2 protein in *LAMP2* KO iPSC-CMs. Left: Western blot image; right: Western blot image merged with gel image indicates the predicted size of the target band; (**G**,**H**) Fast Fourier transformation analysis showed the regularity of α-actinin (**G**) and TNNT2 (**H**) signal distribution is significantly decreased in *LAMP2* KO iPSC-CMs. *N* = 59 for Ctrl, *N* = 73 for *LAMP2* KO. Data from at least 3 independent experiments. Red lines indicate the mean value of each group. *** *p* < 0.001, and **** *p* < 0.0001 versus Ctrl iPSC-CMs by unpaired t-test.

To recapitulate the cardiac phenotype of DD in a iPSC model, we differentiated both Ctrl and isogeneic *LAMP2* KO lines into iPSC-derived cardiomyocytes (iPSC-CMs) using a standardized protocol [25]. Both Ctrl and *LAMP2* KO iPSCs were successfully differentiated into beating iPSC-CMs with no significant difference in efficiency. The iPSC-CMs were characterized with immuno-fluorescent staining of cardiac marker proteins α-actinin and troponin T. While the typical signal pattern of sarcomere protein staining was present in both groups (Figure 2A,C), the *LAMP2* KO iPSC-CMs showed irregular and noisy patterns of fluorescent signal (Figure 2B,D). As expected, both qPCR and Western blot analysis confirmed significant down-regulation of LAMP2 mRNA and absence of LAMP2 protein (~120 kDa) in *LAMP2* KO iPSC-CMs. (Figure 2E,F) Cell morphology analysis has confirmed a slight increase in cell size (Supplemental Figure S2A,B), unchanged perimeter, and more-elongated shape (Supplemental Figure S2C) of *LAMP2* KO iPSC-CMs. Using fast Flourier transformation (FFT) analysis (Supplemental Figure S2D), we found that the regularity of both α-actinin and troponin T staining signal distributions became more disarranged, as evidenced by lower FFT power, in *LAMP2* KO iPSC-CMs. (Figure 2G,H) Additionally, the signal distribution period was increased from the regular physiological range of 1.99 ± 0.07 µm in Ctrl cells to 2.70 ± 0.07 µm in the *LAMP2* KO iPSC-CMs. (Supplemental Figure S2E) The staggered distribution of M band and Z disc protein was also disrupted. (Supplemental Figure S2F).

### 3.3. Blunted Responses of Calcium Handling Regulation to β-Adrenergic Stimulation in LAMP2 KO iPSC-CMs

**Figure 3:**
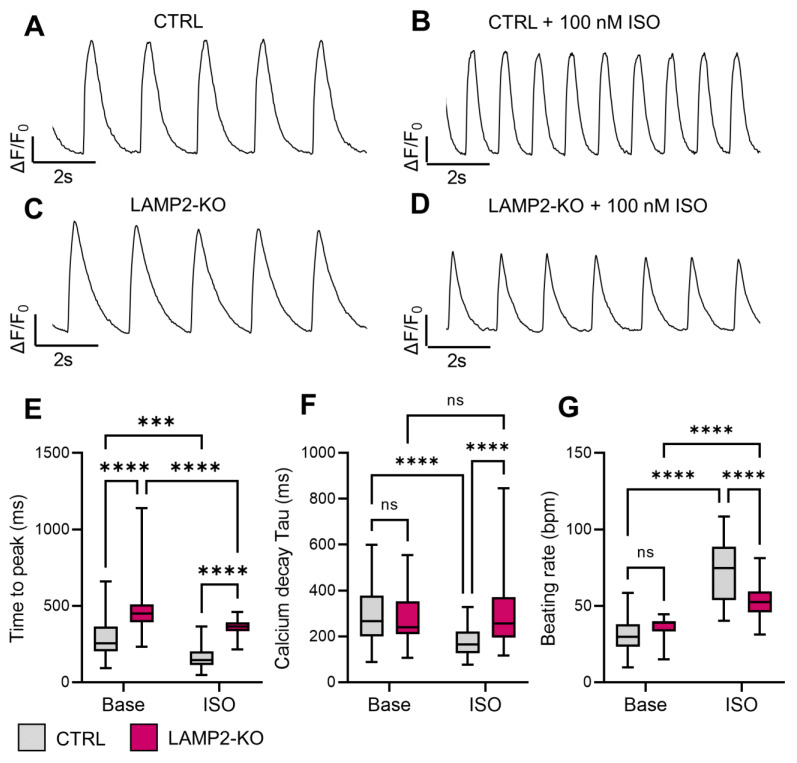
*LAMP2* KO impairs β-adrenergic signaling in iPSC-CMs. (**A**,**B**) Representative Fluo-4 calcium transient trace in Ctrl iPSC-CMs before (**A**) and after (**B**) isoproterenol (ISO) treatment; (**C**,**D**) Representative Fluo-4 calcium transient trace in *LAMP2* KO iPSC-CMs before (**C**) and after (**D**) ISO treatment; (**E**–**G**) *LAMP2* KO iPSC-CMs exhibit prolonged transient rise ti€(**E**) and unchanged transient decay Tau (**F**) and beating rate (**G**) at baseline compared to Ctrl group. ISO treatment induced smaller functional improvement in *LAMP2* KO groups compared to Ctrl, resulting in significant differences between Ctrl and *LAMP2* KO cells. *N* = 59, 103, 37, and 90 cells in Ctrl, *LAMP2* KO, Ctrl + ISO, and *LAMP2* KO + ISO group from at least 3 independent experiments. ns: no significance, *** *p* < 0.001 and **** *p* < 0.0001 by two-way ANOVA test followed by Holm–Sidak method.

Severe cardiac arrhythmia has been reported in DD patients, which can lead to sudden cardiac death, and only heart transplantation can reduce mortality [31,32]. The underlying mechanism of arrhythmogenesis of DD has not been clarified in previous modeling studies [12,17,18]. To systematically understand the cardiac physiological function in our DD model, we first examined the calcium handling in both Ctrl and *LAMP2* KO iPSC-CMs with Fluo-4 AM. Using a high-frame rate live cell imaging platform, the spontaneous calcium transients were recorded in both Ctrl and *LAMP2* KO cells after day 30 of differentiation. (Figure 3A,C) We also treated the cells with a saturated dose of isoproterenol (100 nM ISO) to test the performance of the iPSC-CMs under challenge. (Figure 3B,D) Analysis of the imaging data showed a slight increase in the transient rise time (time to peak) in *LAMP2* KO iPSC-CMs compared to Ctrl (Figure 3E), while the calcium recycling rate (Decay Tau) and the spontaneous beating rate remain unchanged at base level (Figure 3F,G). After the ISO treatment, a significant functional improvement in calcium handling was observed in the Ctrl group in almost all the transient parameters, such as reduced transient rise time, accelerated calcium recycling, and increased beating rate, while the *LAMP2* KO iPSC-CMs only showed much milder, if not any improvement in response to ISO. (Figure 3E–G) All these data indicate that *LAMP2* KO iPSC-CMs exhibit near-normal calcium handling function at the basal level, yet their responsiveness to β-adrenergic stimulation was blunted and could not adjust functional performance upon increased physiological demand. 

### 3.4. Metabolic Maturation Medium Exaggerates the Calcium Handling Abnormalities in LAMP2 KO iPSC-CMs

**Figure 4:**
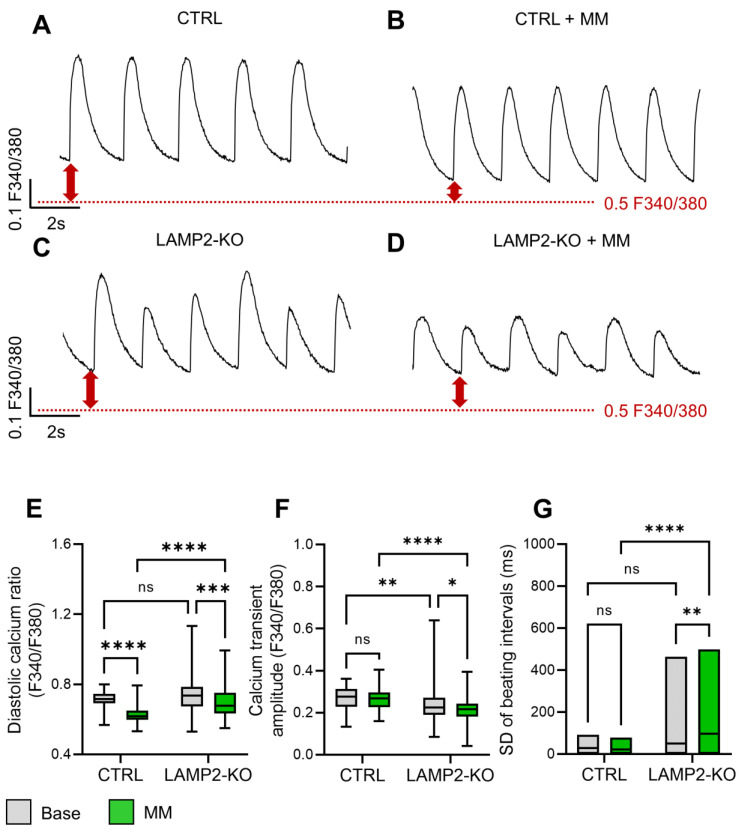
Metabolic maturation enhanced pro-arrhythmic calcium signaling in *LAMP2* KO iPSC-CMs. (**A,B**) Representative Fura-2 calcium transient trace in Ctrl iPSC-CMs before (**A**) and after (**B**) maturation medium (MM) treatment for 48 h; (**C**,**D**) Representative Fura-2 calcium transient trace in *LAMP2* KO iPSC-CMs before (**C**) and after (**D**) maturation medium (MM) treatment for 48 h. Cell were paced at 0.5 HZ in all the groups; (**E**–**G**) *LAMP2* KO iPSC-CMs showed unchanged diastolic cytosol calcium level (**E**), slightly decreased calcium transient amplitude (**F**), and a trend of increased irregular beating (**G**) compared to Ctrl group. MM treatment significantly brought down the diastolic calcium levels in both Ctrl and *LAMP2* KO iPSC-CMs, yet only induced decreased transient amplitude and increased irregular beating events in the *LAMP2* KO group. *N* = 80, 91, 88, and 114 cells in Ctrl, *LAMP2* KO, Ctrl + MM, and *LAMP2* KO + MM group from at least 3 independent experiments. ns: no significance, * *p* < 0.05, ** *p* < 0.01, *** *p* < 0.001, and **** *p* < 0.0001 by two-way ANOVA followed by test Holm–Sidak method.

One major limitation of using iPSCs as cardiac disease models is the immaturity of the differentiated cardiomyocytes [21,33,34,35]. Indeed, most of the iPSC-CMs are differentiated and maintained in RPMI-based medium with [Ca^2+^] of ~0.5 mM, while physiological [Ca^2+^] in human blood is ~1.8 mM. Thus, RPMI based medium will not support the establishment of proper physiological function in iPSC-CMs for disease modeling. Luckily, recent progress in bioengineering and cell biology have identified many approaches to improve the maturity of cardiomyocytes differentiated in vitro [36]. To promote the maturity of our iPSC-CM model for better recapitulation of a DD phenotype, we adopted a metabolic maturation medium (MM) reported by Feyen et al. in our study [22]. Both Ctrl and *LAMP2* KO iPSC-CMs were switched to the MM medium for 2 days, and both groups of cells at base level and after MM treatment were subjected to calcium handling measurement with Fura-2 AM, a ratiometric calcium dye that allows the evaluation of both diastolic calcium and calcium dynamics. (Figure 4A–D) To avoid the variation caused by different beating rates, all the experiments was performed under electrical stimulation at 0.5 HZ as optimized in our previous study [29]. Calcium recording showed only a minor difference between Ctrl and *LAMP2* KO at the basal level, as there was no significant change in the diastolic calcium and the standard deviation of beating intervals (indicates the pro-arrhythmic irregular beatings). There was only a slight decrease in the transient amplitude in *LAMP2* KO cells. (Figure 4E–G) The treatment of MM medium seemed to improve the calcium handling in general, as both Ctrl and *LAMP2* KO groups showed decreased diastolic calcium, and faster calcium recycling (Figure 4E). However, the MM exaggerated the DD phenotype in *LAMP2* KO iCMs, as they showed overloaded diastolic calcium, lower calcium amplitude, and much increased arrythmia events compared to Ctrl cells after treatment (Figure 4E–G).

### 3.5. Metabolic Maturation Promotes ROS Overload and CaMKII Activation in LAMP2 KO CMs

**Figure 5:**
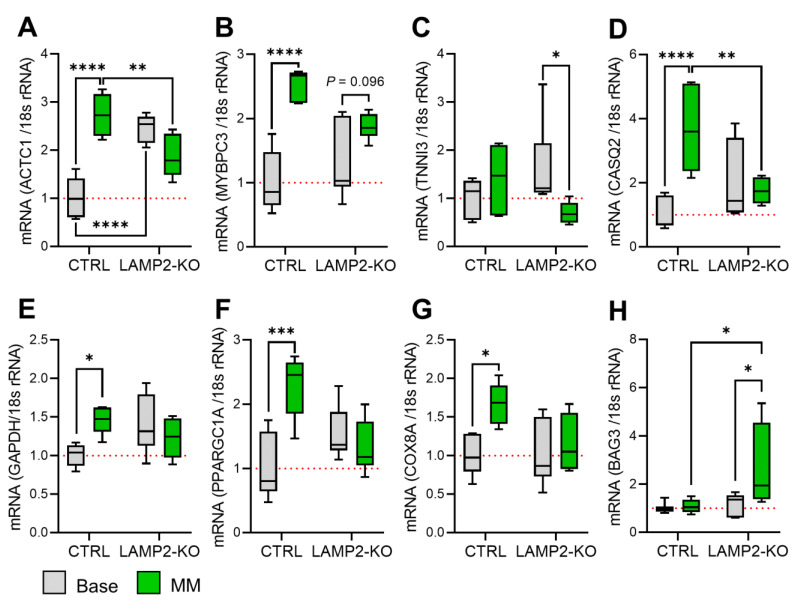
Real-time PCR quantification of mRNA expression of key cardiac genes in Ctrl and *LAMP2* KO iPSC-CMs. (**A,B**) Increased expression of sarcomere and cytoskeletal genes ACTC1 (**A**) and MYBPC3 (**B**) genes in the Ctrl but not *LAMP2* KO iCMs after MM treatment; (**C**) Decreased expression of adult troponin I subtype, TNNI3, in MM-treated *LAMP2* KO cells; (**D**) Increased expression of CASQ2, which encodes Ca^2+^ buffering protein calsequestrin-2 in the Ctrl but not *LAMP2* KO iCMs after MM treatment; (**E**–**G**) Increased mitochondrial gene expression, such as GAPDH (**E**), PPARGC1A (**F**), and COX8A1 (**G**) in Ctrl but not *LAMP2* KO cells after MM treatment; (**H**) Increased expression of BAG3, a cochaperone protein of HSP70, in *LAMP2* KO but not Ctrl iCMs after MM treatment. Results from at least 3 independent experiments. Red dashed line indicates the relative mRNA expression in Ctrl group at base level were normalized to 1. * *p* < 0.05, ** *p* < 0.01, *** *p* < 0.001, and **** *p* < 0.0001 by two-way ANOVA test followed by Holm–Sidak method.

**Figure 6:**
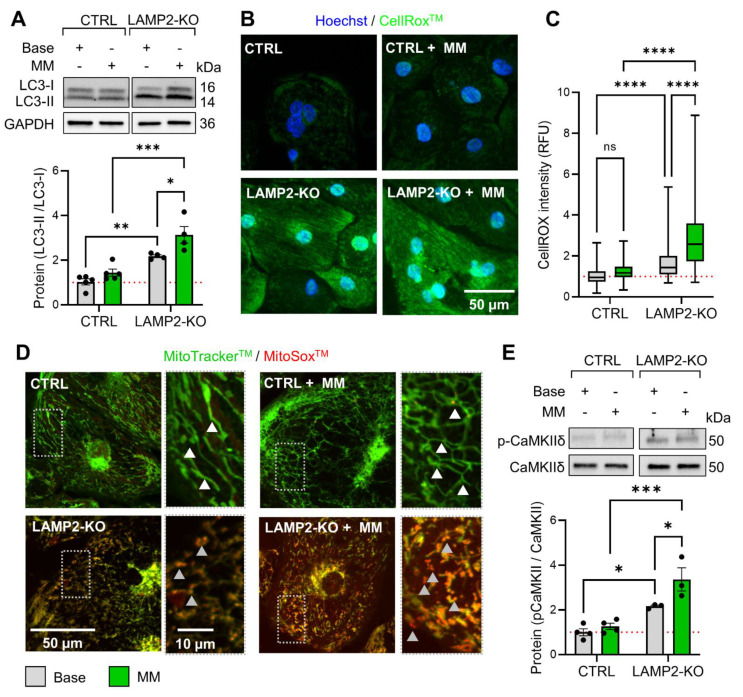
ROS overload led to over-activation of CaMKII in *LAMP2* KO iCMs after MM treatment. (**A**) Western blot quantification of LC3-I and LC3-II expression in Ctrl and *LAMP2* KO iCMs. Impaired autophagic flux is evidenced by the significantly increased LC3-II/I ratio, as the degradation mechanism of LC3-II is compromised in *LAMP2* KO both before and after MM treatment. Results from at least 4 independent experiments; (**B**) Representative images of Hoechst and CellRox^TM^ staining of cellular ROS levels; (**C**) Quantitative results showed increased ROS level in *LAMP2* KO and *LAMP2* KO + MM groups. *N* = 147, 167, 182, and 136 cells in Ctrl, *LAMP2* KO, Ctrl + MM, and *LAMP2* KO + MM group from at least 3 independent experiments. ns: no significance; (**D**) Representative images of MitoTracker^TM^ green and MitoSox^TM^ red co-staining of mitochondrial morphology and mROS levels. Rectangular images showed zoom-in views of the dashed boxes. White arrows indicate the mitochondrial networks. Grey arrows indicate fragmentized mitochondria with increased mROS levels; (**E**) Western blot quantification of the expression and phosphorylation levels of cardiac CaMKIIδ. The p-CaMKIIδ/CaMKIIδ ratio is significantly increased in *LAMP2* KO and *LAMP2* KO + MM groups. Results from at least 3 independent experiments. Red dashed line indicates the value in Ctrl group at base level were normalized to 1. * *p* < 0.05, ** *p* < 0.01, *** *p* < 0.001, and **** *p* < 0.0001 by two-way ANOVA test followed by Holm–Sidak method.

To understand the molecular basis of MM treatment related arrythmia in *LAMP2* KO CMs, we first compared the mRNA expression of cardiac function related genes in Ctrl and *LAMP2* KO CMs by real-time PCR. In our results, the expression of many cytoskeleton genes was increased in Ctrl cells upon MM treatment, which is in line with a higher demand for contractility (Figure 5A,B) Yet, this trend could not be observed in *LAMP2* KO cells, as the expression of the mature subtype of TNNI3 even decreased after MM treatment. (Figure 5C) The calcium handling proteins (such as ATP2A2, RYR2, CACNAIC, and PLN, etc.), except CASQ2, remained unchanged in both Ctrl and *LAMP2* KO groups. (Figure 5D, Supplemental Figure S3A–D) Interesting, the mitochondrial proteins, such as GAPDH and PPARGC1A, were significantly upregulated in the Ctrl group after MM treatment, but not in the *LAMP2* KO group, indicating that MM treatment may induce increased autophagic stress in the KO cells, thus failed to promote metabolic maturation of mitochondria. (Figure 5E–G) This hypothesis is supported by the increased expression of protein homeostasis related genes and the stress maker NPPA specific to the *LAMP2* KO cells after MM treatment, (Figure 5H, Supplemental Figure S3E–H) although other mitochondrial and sarcomere proteins remained unchanged (Supplemental Figure S3I–L). To further confirm the hypothesis, we measured the autophagic marker LC3II/I ratio by Western blot. Our result confirmed that LC3II/I ratio is unchanged in MM-treated Ctrl cells, yet due to the absence of LAMP2, KO cells already showed an increased LC3II/I ratio at baseline, which was further increased by MM treatment. (Figure 6A) As autophagy controls the renewal of mitochondrial through mitophagy, impaired autophagy flux may lead to accumulation of dysfunctional mitochondrial and increase of cytosolic and mitochondrial ROS. Indeed, our follow-up CellROX and MitoSox measurements confirmed ROS overload in the cytosol and mitochondria of *LAMP2* KO cells, both before and after MM treatment. (Figure 6B–D, Supplemental Figure S4A) Moreover, the mitochondria in *LAMP2* KO cells were damaged by the lack of autophagic flux and MM challenge, as evidenced by more fragmentized morphology and shortened branch length compared to Ctrl cells. (Supplemental Figure S4B,C) As a result of increased ROS, the phosphorylation of the main cardiac CaMKII subunit, CaMKIIδ, was greatly increased in *LAMP2* KO CMs compared to Ctrl group, which is known to be a key kinase that promotes arrhythmia in the heart (Figure 6E).

### 3.6. ROS Scavenger Restored Calcium Homeostasis in LAMP2 KO Cells 

**Figure 7:**
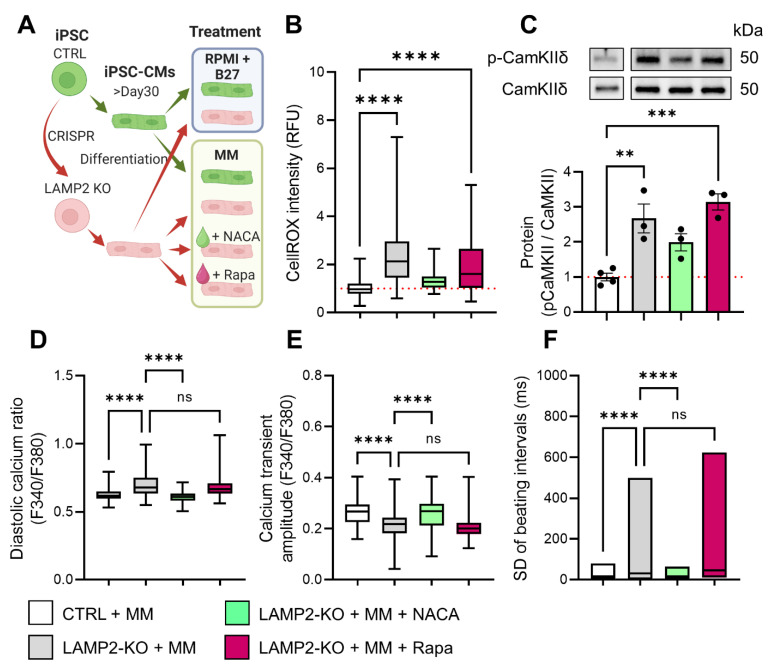
Restoration of calcium homeostasis of *LAMP2* KO iCMs by ROS scavenger. (**A**) Diagram of experimental design. (**B**) Treatment of NACA but not rapamycin significantly reduced the cellular ROS overload in *LAMP2* KO iCMs in MM. *N* = 181, 135, 145, and 143 cells in Ctrl + MM, *LAMP2* KO + MM, *LAMP2* KO + MM + NAC, and *LAMP2* KO + MM + Rapa group from at least 3 independent experiments. (**C**) Western blot showed NACA reduced the p-CaMKIIδ/CaMKIIδ ratio in *LAMP2* KO iCMs, but no change was detected in rapamycin treated cells. Results from at least 3 independent experiments. (**D**–**F**) Fura-2 calcium imaging analysis suggested that NACA treatment restored the diastolic calcium level (**D**), calcium transient amplitude (**E**), and regular beating (**F**) in *LAMP2* KO iCMs, while no beneficial effect was observed in the rapamycin treated group. *N* = 88, 114, 105, and 98 cells in Ctrl + MM, *LAMP2* KO + MM, *LAMP2* KO + MM + NAC, and *LAMP2* KO + MM + Rapa group from at least 3 independent experiments. Red dashed line indicates the value in Ctrl group at base level were normalized to 1. ns: no significance, ** *p* < 0.01, *** *p* < 0.001, and **** *p* < 0.0001 by one-way ANOVA test followed by Tukey’s test.

**Figure 8:**
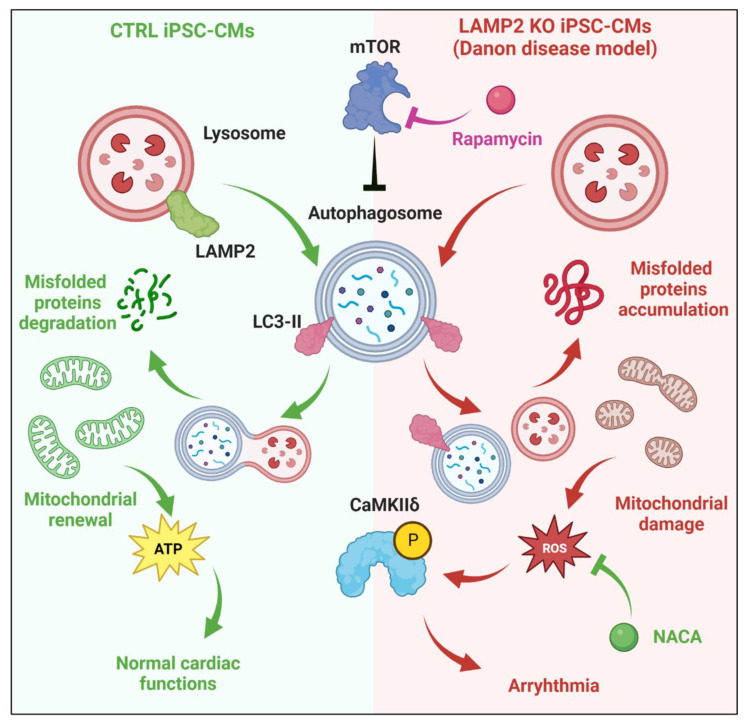
Diagram of the cellular mechanisms of arrhythmogenesis in DD cardiomyocytes. Left: in healthy cardiomyocytes, active autophagy mechanisms maintain the protein homeostasis and mitochondrial function. Right: in Danon disease, the lack of LAMP2 impaired the maturation of lysosome and its fusion with autophagosome, result in the accumulation of misfolded proteins and damaged mitochondria. Moreover, the increased demand of contractile function in response to increased adrenaline level (in patients) or to MM (in iPSC-CM model) will exaggerate the autophagic stress. Damaged mitochondria lead to ROS overload and overactivation of CaMKIIδ, which is known to be a key regulator during the pathogenesis of cardiac remodeling and arrhythmia.

As we have identified ROS-CaMKII as a key signaling event during the pathogenesis of *LAMP2* KO CMs, next we sought to test whether getting rid of excess ROS load eliminates the pro-arrhythmic events in calcium handling. For that, we treated *LAMP2* KO cells exposed to MM with ROS scavenger NACA (*N*-acetylcysteine amide) to test the beneficial effect on them. Rapamycin, a well-known autophagy activator, was used as a control. (Figure 7A) Our results showed that NACA significantly reduced ROS to the level of the Ctrl group in both cytosol and mitochondria. (Figure 7B, Supplemental Figure S5A) In comparison, rapamycin was not able to affect the ROS accumulation in mitochondria, while only partially reducing ROS in the cytosol, which may be due to other signaling pathways that are independent of autophagic regulation [37,38]. As a result, the p-CaMKIIδ level was also significantly reduced in NACA-treated, but not in rapamycin-treated cells. (Figure 7C) Finally, we evaluated the calcium handling in both Ctrl and *LAMP2* KO cells with Fura-2 calcium imaging. Our results showed NACA treatment restored calcium homeostasis in *LAMP2* KO cells upon MM treatment, while no improvement was observed for the rapamycin group. (Figure 7D–F) Importantly, further experiments showed NACA treatment could not bring down the LC3II/I ratio in *LAMP2* KO cells (Supplement Figure S5B), indicating the ROS scavenger exerts its beneficial effect on *LAMP2* KO cells through targeting ROS overload and arrythmia, rather than fixing the impaired autophagic mechanism (Figure 8).

## 4. Discussion

### 4.1. Functional Maturation of iPSC-CMs and Modeling of DD

It is known that there are three LAMP2 isoforms in human due to alternative splicing [39]. LAMP2A is involved in chaperone-mediated autophagy (CMA), while LAMP2B is primarily related to macroautophagy, a key mechanism regulating protein homeostasis in the most energy-consuming organs such as heart, skeletal muscle, and brain [5,40,41,42]. LAMP2C is predicated to be important for the autophagic degradation of RNA and DNA [43,44]. Although DD results in the deficiency of all 3 isoforms of LAMP2, DD mutations were only identified in LAMP2B, indicating the key role of this isoform in the causality of DD [16,45,46]. A functional heart needs to pump blood constantly, which is heavily reliant on the dynamic regulation of protein homeostasis and the integrity of cardiac contractile mechanisms, such as the sarcomere. However, similar to the DD heart, the absence of LAMP2 protein in our DD iPSC-CM model leads to impaired lysosome maturation and deficiency of autophagic flux. As a result, misfolded proteins in the cardiomyocytes cannot be degraded properly and will accumulate in lysosomal vacuoles, and the sarcomere structure becomes disarranged due to the failure of protein quality control mechanisms. (Figure 2) Like previous reports, treatment of control iPSC-CMs with MM, which was supplemented with essential ingredients for cardiac muscle growth and metabolic maturation, such as fatty acids, triiodothyronine, glucocorticoid, and insulin-like growth factor 1(IGF1), etc., promoted the functional maturation of iCMs, as the Ctrl cells displayed faster calcium recycling, increased calcium dynamics, and stronger contractile force. (Figure 4) [47,48,49,50,51]. However, the treatment of *LAMP2* KO iCMs with MM aggravated the DD phenotype. This is because improved cardiac function is based on robust regulation of protein homeostasis, which is deficient in *LAMP2* KO cells. As a result, more misfolded proteins will aggregate in the *LAMP2* KO iCMs upon MM treatment. This was confirmed by our LC3 Western blot, in which MM treated *LAMP2* KO iCMs showed the highest LC3 II/I ratio. As a result, increased autophagic stress led to more severe phenotype in our MM treated *LAMP2* KO DD model. 

### 4.2. Cellular Mechanism of Arrhythmia in DD Cardiomyocytes

About 96.2% of hemizygous male patients of DD are affected by early onset severe hypertrophic cardiomyopathy (HCM), which is characterized by cardiac hypertrophy, thickening of ventricle walls, and heart failure [9,11]. Besides the morphological remodeling of the heart, ECG abnormalities are also highly prevalent in DD patients. It is estimated that heart block presents in ~ 35–50% of DD patients and requires pacemaker implantation. Moreover, about 60% of DD patients were diagnosed with life-threatening atrial fibrillation and ventricular arrhythmias, which lead to sudden cardiac death of DD patients [31,32]. It is believed that DD heart arrhythmias likely result from either increased cardiac cell death and fibrosis or glycogen-filled myocytes (due to the lack of autophagic flux), both of which provide the anatomical basis for the interruption of electrical conduction in the heart [9]. However, the cellular mechanisms of the arrhythmogenesis in DD cardiomyocytes remains unclear. In our study of the *LAMP2* KO DD iPSC-CM model, we have identified a deficiency of autophagy signaling and increased oxidative stress, especially in response to functional maturation (MM treatment). (Figure 5A–D) This result is in line with a previous report in DD patient-specific iPSC-CMs that carry *LAMP2* mutations [18]. Cellular oxidative stress may be partially due to autophagic and mitophagic deficiency related to mitochondrial dysfunction, which is confirmed by our observed mitochondrial morphologic remodeling and mROS overload [52]. Increased oxidative stress is related to multiple types of damage in cardiomyocytes, such as impaired contractile force, decreased SERCA2a activity, loss of intercellular calcium homeostasis, and metabolic dysfunction [53,54]. Importantly, recent studies have shown that Ca^2+^/calmodulin dependent protein kinase II (CaMKII) as a sensor of cellular ROS via direct oxidation of its regulatory domain, which lead to the activation of CaMKII in the absence of Ca^2+^/CaM [55,56]. Furthermore, excess CaMKII activation has been associated with cardiac arrhythmias such as atrial fibrillation. Indeed, our results have identified the over-activation of CaMKII in MM treated *LAMP2* KO DD models, confirming the potential role of CaMKII oxidation and activation during the pathogenesis of arrhythmia in DD patients. Interestingly, CaMKII oxidation in DD is not dependent on acute or prolonged β-adrenergic receptor signaling, indicating that CaMKII activation may occur even before the onset of significant cardiac functional deficiency in the heart of DD patients. Although we demonstrated novel pathological mechanisms of arrythmia in DD, it’s important to note that the current study is mainly based on cellular models, thus lack the contexts of intercellular interactions at tissue level. Thus, our disease model may not recapitulate other key factors that are known to contribute to the arrythmia in DD, such as increased apoptosis and fibrosis, as well as the blockade of electrical conductions, etc. To better understand these etiologies of DD at a higher level, iPSC based engineered heart tissue (EHT) and cardiac organoid are promising modeling systems. 

### 4.3. Outlook for the Future Treatment of DD

Due to incomplete understanding of the molecular mechanism of DD, our current treatments are mostly designed to alleviate disease phenotypes [57]. Standard guidelines for hypertrophic cardiomyopathy and heart failure will be followed for the treatment of DD patients. For example, in the DD patients with cardiac pre-excitation and arrhythmia, ablation therapy will be also considered to prevent sudden cardiac death [9]. As LAMP2 deficiency is the central cause of the DD, multiple new therapies are now being developed to correct LAMP2 function. For example, Chi et al. used CRISPR-Cas9 technology to correct the point mutation in the DD patient-iPSC-CMs and was able to improve metabolic function [16]. Additionally, inducing sustained LAMP2B expression in *LAMP2* KO mice using recombinant adeno-associated virus 9 (AAV9) restored cardiac function and survival rates [58]. In addition to gene therapies, small molecules are also being tested for therapeutic applications. For example, the mammalian target of rapamycin (mTOR) is known as a key regulator of macroautophagy, and its inhibition of mTOR by rapamycin or metformin signaling are known to exert beneficial effects in cardiac disease [59]. In LAMP2 deficiency zebrafish models, mTOR inhibition has been shown to be effective in rescuing the cardiac phenotypes [13]. Moreover, Ng et al. has used the small molecule 5AdC to induce reactivation of the silenced X chromosome in female DD patient-specific iPSC-CMs, which carry heterozygous *LAMP2* mutations. Their results indicate even minor reactivation of LAMP2 expression from a wild type allele can significantly improve autophagy and cardiac function [60]. However, for male DD patients who carry hemizygous *LAMP2* mutations with more severe phenotype, mTOR signaling inhibition may not work, as it is upstream of the pro-autophagic pathway, while the completely loss of functional LAMP2 will not support the improvement of autophagic flux. Indeed, our results demonstrated rapamycin treatment in *LAMP2* KO iPSC-CMs was not able to rescue the cardiac phenotype. (Figure 7D–F) However, treatment of the diseased cells with NAC, the ROS scavenger, significantly reduced the ROX overload in the cytosol and mitochondria and alleviated the pro-arrythmia phenotype in the *LAMP2* KO iPSC-CMs in MM medium, although it could not rescue autophagy function. (Supplemental Figure S4B) These results suggest that autophagic deficiency (increased LC3II/I ratio, sarcomere disarrangement) and arrhythmogenic calcium activities (calcium handling abnormalities and irregular beating) are separately regulated in the DD cardiomyocytes, and our data indicated a potential therapeutic target to prevent arrythmia and sudden cardiac death in DD patient through targeting the overload of ROS and excess activation of CaMKII. 

## 5. Conclusions

In summary, the current work establishes *LAMP2* KO iPSC-CMs for the modeling and study of the pathologic mechanism of Danon disease. Our results confirm that impairment of autophagy in CMs lead to hypertrophic cardiac cells and disarrangement of sarcomeres. Importantly, we identify the mitochondrial morphological change, ROS and mROS overload, CaMKIIδ over-activation, and abnormal calcium handling as key signaling events in *LAMP2* KO iPSC-CMs, suggesting a new mechanism that contributes to DD heart arrhythmogenesis. The current study not only improves our understanding of the pathologic mechanisms of how LAMP2 deficiency contributes to cardiac phenotypes but also provides novel therapeutic targets to prevent arrythmia in DD patients. 

## Data Availability

The data presented in this study are available on request from the corresponding author.

## Supplementary Materials

The following are available online at https://www.mdpi.com/article/10.3390/biom13010069/s1, Figure S1: CRISPR KO design and results for the second *LAMP2* KO iPSC line; Figure S2: Morphological measurement of Ctrl and *LAMP2* KO iPSC-CMs; Figure S3: Re-al-time PCR quantification of mRNA expression of additional key cardiac genes in Ctrl and *LAMP2* KO iPSC-CMs; Figure S4: MM treatment induces morphological and function remodeling of mitochondria in *LAMP2* KO iPSC-CMs; Figure S5: Beneficial effect of ROS scavenger in *LAMP2* KO iPSC-CMs; Table S1: Summary of the sequences of qPCR DNA oligos used in the current study; Table S2: Summary of the information of antibodies used in the current study. Table S3: Summary of the sequences of sgRNA off-target genotyping primers used in the current study.
