# Supplementary material for: Metabolic Maturation Exaggerates Abnormal Calcium Handling in a Lamp2 Knockout Human Pluripotent Stem Cell-Derived Cardiomyocyte Model of Danon Disease
Source: Biomolecules. 2022 Dec 29;13(1):69. doi: 10.3390/biom13010069 (PMC9855424; doi:10.3390/biom13010069)
Supplement: Supplementary file 1 [file biomolecules-13-00069-s001.zip › biomolecules-2128418-supplementary figures.pdf]

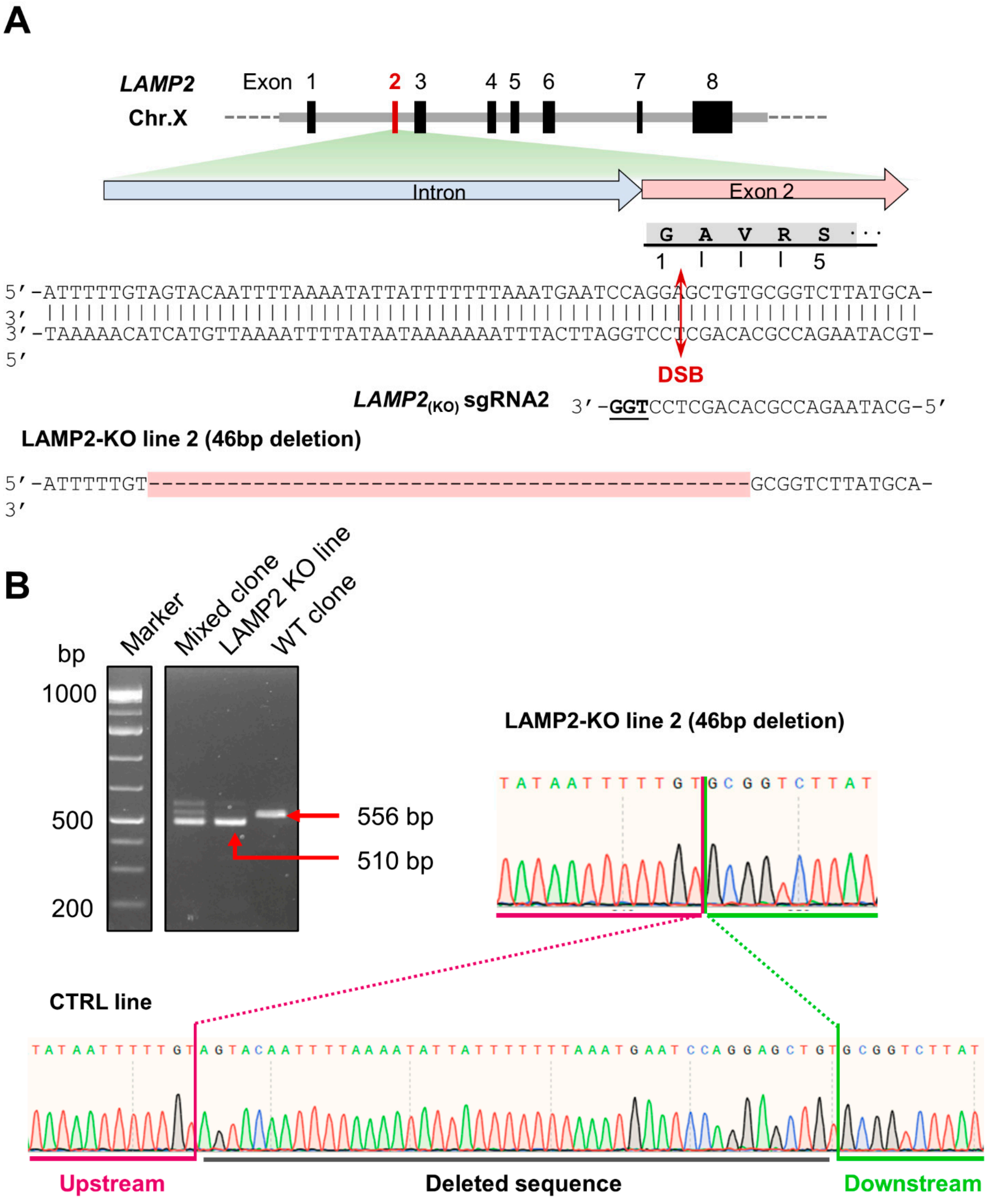

Figure S1. CRISPR KO design and results for the second *LAMP2* KO iPSC line. A. Design of CRISPR KO sgRNA targeting the 2nd exon of *LAMP2*. B: sequencing result confirmed a 46 bp deletion around the splicing site of the *LAMP2* exon 2, which result in the truncation of LAMP2.

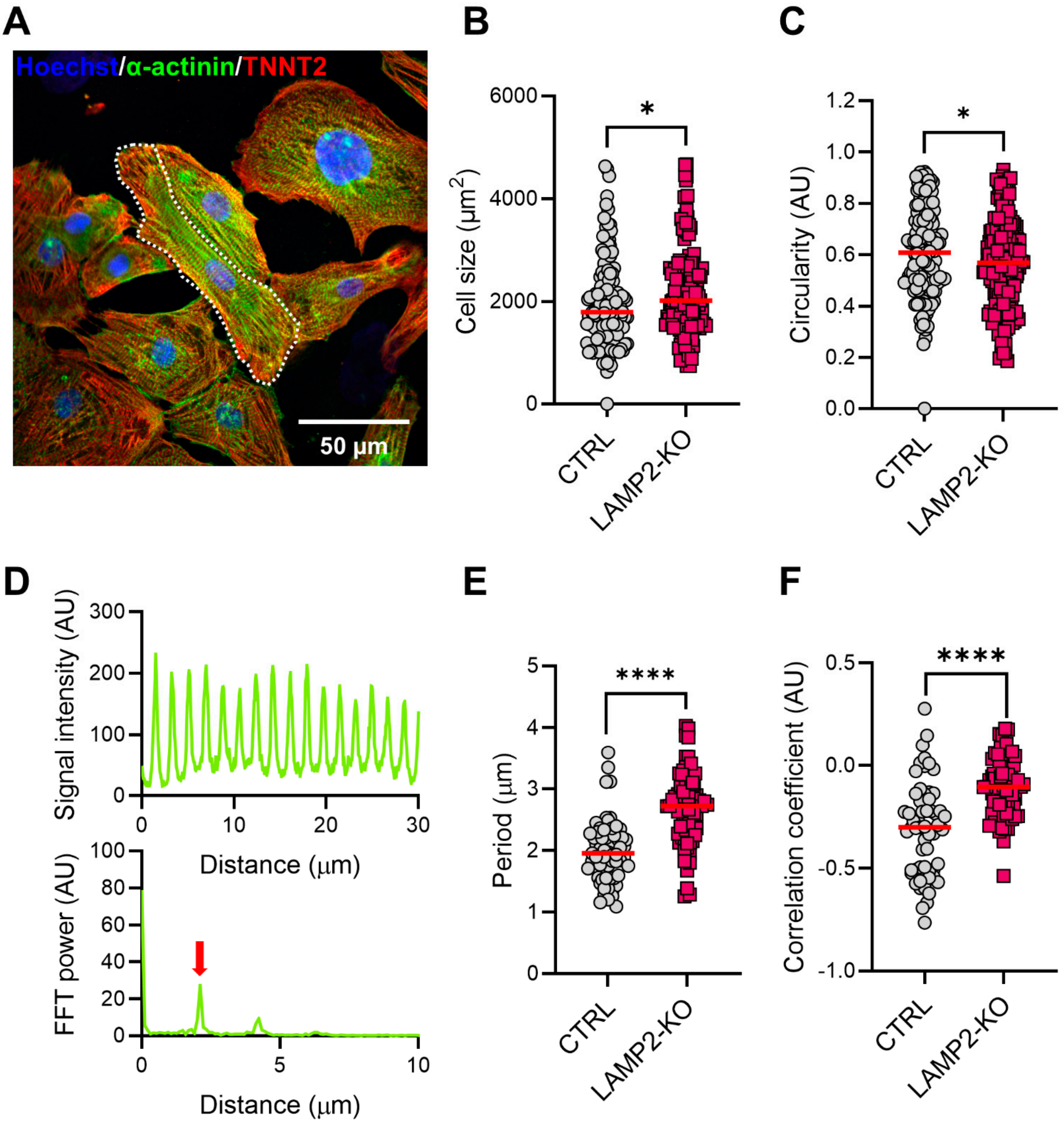

Figure S2. Morphological measurement of Ctrl and *LAMP2* KO iPSC-CMs. A. Representative immuno-fluorescence imaging of *LAMP2* KO iPSC-CMs. Blue: Hoechst; Green: α-actinin; Red: TNNT2. B and C. The *LAMP2* KO iPSC-CMs exhibit enlarged cell size (B) and elongated shape (C) compared to Ctrl cells. N = 136 and 162 cells for Ctrl and *LAMP2* KO group from at least 3 independent experiments. D. Representative traces show the distributions of α-actinin protein signal in immunofluorescence and the frequency distribution of signals after Fast Fourier transformation (FFT). Arrow indicates the peak of signal regularity, which represents the average sarcomere length. E and F. The period of sarcomere signal distribution and the correlation coefficient of α-actinin and TNNT2 signals in Ctrl and *LAMP2* KO iPSC-CMs. N = 59 for Ctrl, N = 73 for *LAMP2* KO. Data from at least 3 independent experiments. *p<0.05 and ****p<0.0001 versus Ctrl iPSC-CMs by unpaired t-test.

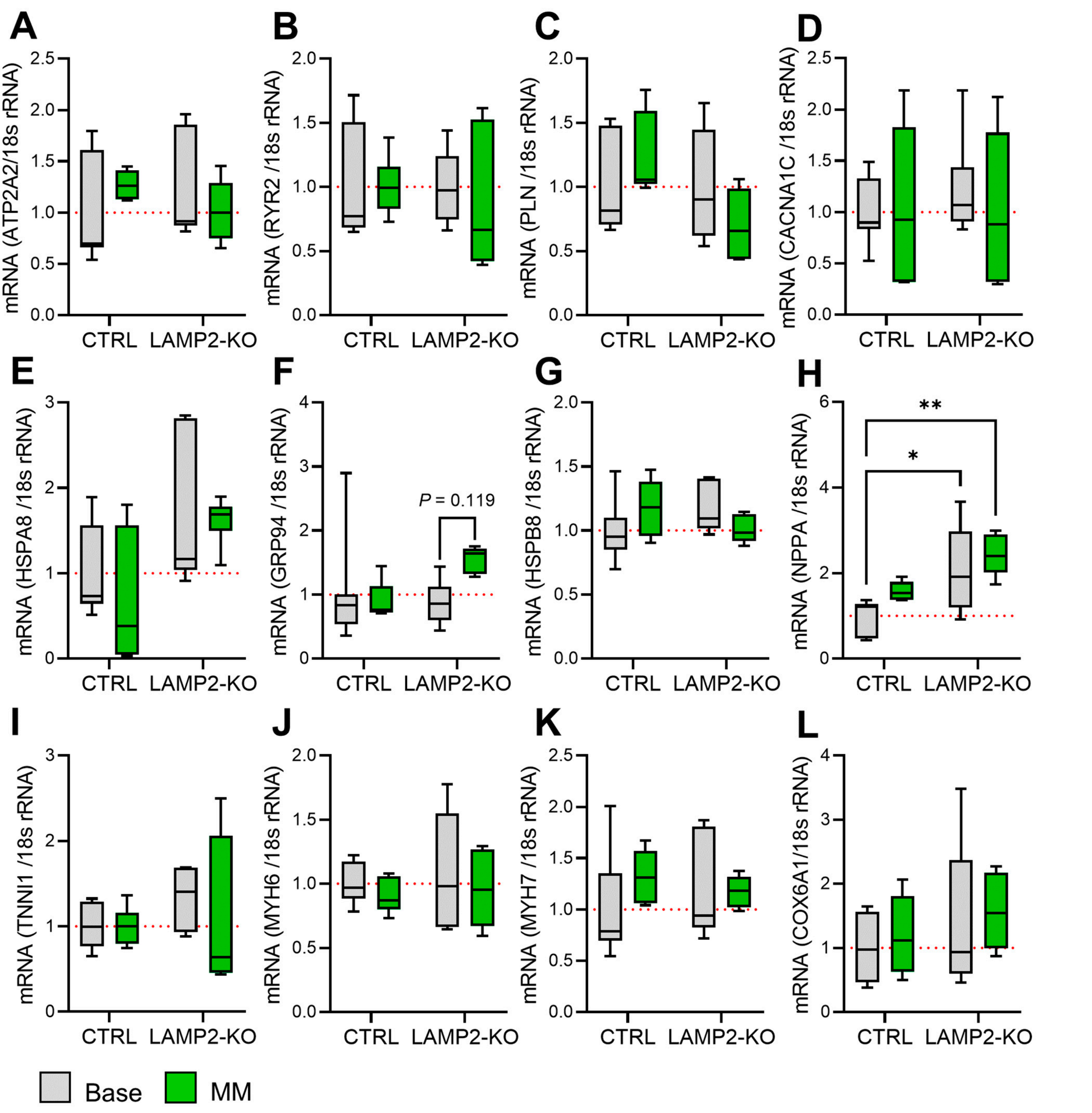

Figure S3. Real-time PCR quantification of mRNA expression of additional key cardiac genes in Ctrl and *LAMP2* KO iPSC-CMs. A–D. There is no significant change of calcium handling gene expression, such as ATP2A2 (SERCA2, A), RYR2 (ryanodine receptor 2, B), PLN (phospholamban, C), and CACNA1C (L-type calcium channel, D) in Ctrl and *LAMP2* KO groups. E to G. Expression of heat shock proteins HSP70 (HSPA8, E) and chaperone protein GRP94 (F) showed trends of upregulation in *LAMP2* KO iPSC-CMs, while no change was observed for heat shock protein HSP22 (HSPB8, G). H. *LAMP2* KO iPSC-CMs showed much higher expression of cardiac stress marker NPPA in both base and MM treated groups. I to K. MM treatment didn't affect the expression of immature subtype of sarcomere proteins TNNI (I) and MYH6 (J), yet the expression of mature subtype sarcomere protein MYH7 (K) showed a trend of upregulation in both groups after MM treatment. L. Mitochondrial protein Cytochrome c oxidase 6A1 (COX6A1) showed a trend of upregulation in MM treated *LAMP2* KO cells. For all the qPCR experiments here, data from at least 3 independent experiments. *$p<0.05$ and **$p<0.01$ versus Ctrl iPSC-CMs by two-way ANOVA test followed by Holm–Sidak method.

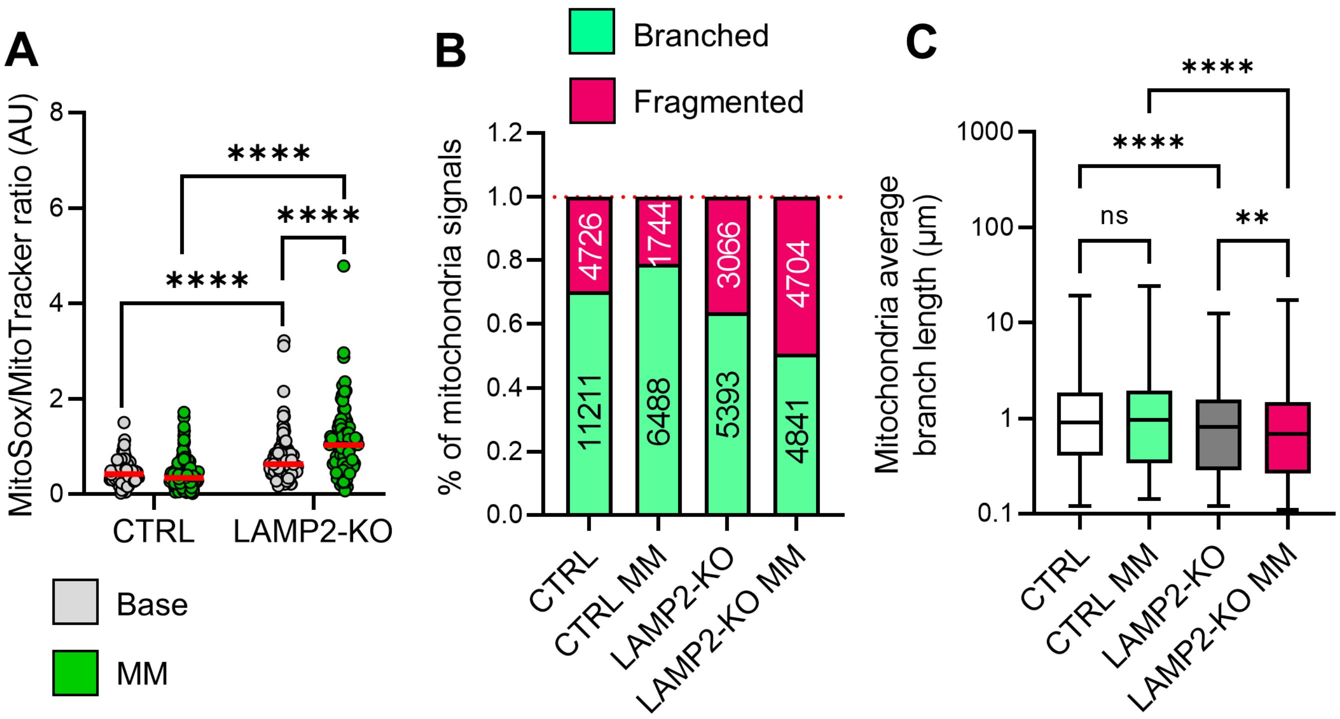

Figure S4. MM treatment induces morphological and function remodeling of mitochondria in *LAMP2* KO iPSC-CMs. A. Mitochondrial ROS levels (mROS) measured as MitoSOX/MitoTracker ratio was unchanged in Ctrl cells upon MM treatment but was greatly increased in *LAMP2* KO cells. N = 151, 223, 150, 112 cells in Ctrl + MM, *LAMP2* KO + MM, *LAMP2* KO + MM + NAC, and *LAMP2* KO + MM + Rapa group from at least 3 independent experiments. ***p<0.001 and ****p<0.0001 versus Ctrl iPSC-CMs by 2-way ANOVA test followed by Holm–Sidak method. B. MM treatment caused an increased percentage of fragmented mitochondria in *LAMP2* KO iPSC-CMs but not in Ctrl iPSC-CMs. C. The average length of mitochondrial branches was decreased in LAMP2 KO iPSC-CMs compared to Ctrl group, both before and after MM treatment. N = 11211, 6488, 5393, 4841 branch counts in Ctrl + MM, *LAMP2* KO + MM, *LAMP2* KO + MM + NAC, and *LAMP2* KO + MM + Rapa group from at least 3 independent experiments. **p<0.01 and ****p<0.0001 by one-way ANOVA test followed by Tukey's test.

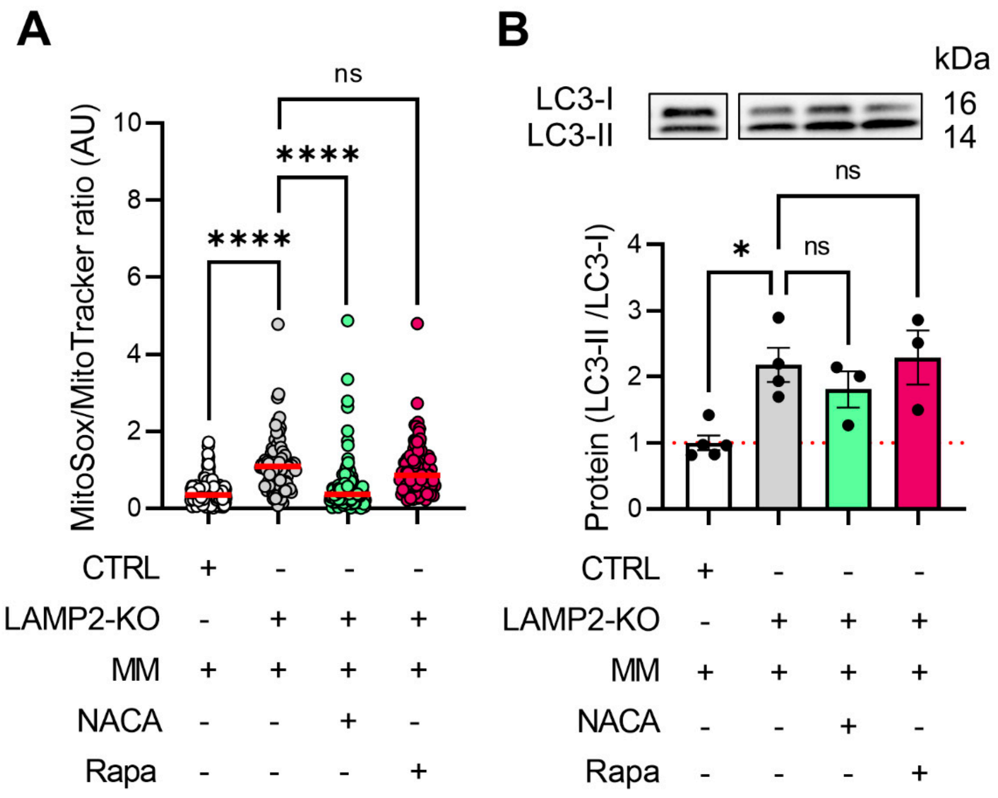

Figure S5. Beneficial effect of ROS scavenger in *LAMP2* KO iPSC-CMs. A. Treatment of NACA but not rapamycin in *LAMP2* KO cells is able to restore the mROS level under MM treatment. N = 223, 112, 170, 103 cells in Ctrl + MM, *LAMP2* KO + MM, *LAMP2* KO + MM + NAC, and *LAMP2* KO + MM + Rapa group from at least 3 independent experiments. ****p<0.0001 by 1-way ANOVA test followed by multiple comparison method. B. Western blot quantification of LC3II/I ratio showed that neither NACA nor rapamycin treatment was able to rescue the impaired autophagic flux in the *LAMP2* KO iPSC-CMs. Data from at least 3 independent experiments. **p<0.01 by one-way ANOVA test followed by Tukey's test.
