# Supplementary material for: Metabolic Maturation Exaggerates Abnormal Calcium Handling in a Lamp2 Knockout Human Pluripotent Stem Cell-Derived Cardiomyocyte Model of Danon Disease
Source: Biomolecules. 2022 Dec 29;13(1):69. doi: 10.3390/biom13010069 (PMC9855424; doi:10.3390/biom13010069)
Supplement: Supplementary file 1 [file biomolecules-13-00069-s001.zip › Supplemental Table S1.pdf]

Supplemental Table S1. Summary of the sequences of qPCR oligos used in the current study.

Gene name	Primer sequences		Function
	Forward	Reverse	
**Target protein**			
LAMP2	GGGTTCAGCCTTTCAATGTGAC	GCACAGCATGTCCTGGCT	lysosomal associated membrane protein 2
**Calcium handling**			
ATP2A2	ATGGGGCTCCAACGAGTTAC	ACACCCACAATTGCATTGGC	SERCA sarco/endoplasmic reticulum Ca2+-ATPase
CASQ2	AGGAACACCAAAGACCCACTC	GATGCTCAGATCGGGGTTGT	calsequestrin2
RYR2	CGTGTGGGTCGGAGACATTA	AGAGATTGACAGCCCGATGC	ryanodine receptor 2
PLN	CCAGTCAAACACCCGTAAGACT	GAGCGAGTGAGGTATTGGACT	phospholamban
CACNA1C	AGATGACTGCTTATGGGGCTT	CACTGAACCACATGCTTTAGCC	calcium voltage-gated channel subunit alpha 1 C
SCN5A	CAGCAGTGGAAAGCGGAAC	AGTCCTCTGGGTCCTTGACA	sodium voltage-gated channel alpha subunit 5
**Sarcomere**			
MYH6	TGGACAAGCTGCAACTGAAGG	AGTGTCACTCCTCATCGTGCAT	myosin heavy chain 6
MYH7	GCTCTGTGTCTTTCCCTGCT	TGAGGTCAAAAGGCCTGGTC	myosin heavy chain 7
TNNI1	CTCCCGCAAACTCTTGCTGA	GGTGTTGTGGAGGCATTTGG	troponin I1
TNNI3	CAAAGCAAGAGCTGGAGCGA	TCAGATCTGCAATCTCCGTGA	troponin I3
MYBPC3	CATGAGGCGCGATGAGAAGA	ATGTACTTGCTGCCGCTCAT	myosin binding protein 3C
ACTC1	GCCGACGAACCCCCT	CGTAGGAGTCCTTCTGACCC	actin alpha cardiac muscle 1
**Autophagy and protein homeostasis**			
BAG3	CCCCGTTCAGGTCATCTGTC	TTTCTCGATGGGTCATGGGC	BAG cochaperone 3
HSPA8	TATTGGAGCCAGGCCTACAC	TCTCGACTTTTCCGTGCTGG	heat shock protein (Hsp70) family A member 8
HSPB8	CAGCAAGAAGGTGGCATTGTT	GGGGAAGCTCGTTGTTGAAA	heat shock protein family B (small) member 8
HSP90B1	TCTAGGACGGGGAACGACAA	TGGGCTCCTCAACAGTTTCAG	heat shock protein 90 beta family member 1
**Metabolism**			
GAPDH	GGAGCGAGATCCCTCCAAAAT	GGCTGTTGTCATACTTCTCATGG	glyceraldehyde-3-phosphate dehydrogenase
PPARGC1A	CAAGCAAAGGGAGAGGCAGA	TTCGTTTGACCTGCGCAAAG	PPARG coactivator 1 alpha
COX8A	TGTGGTGTACTCCGTGCCAT	GGTCACGAAGCAGGAGGTAA	cytochrome c oxidase subunit 6A1
COX6A1	CATCTCCGCATCAGGACCAA	ATGTGCAGAGTAACGGTCCA	cytochrome c oxidase subunit 8A
**Stress marker**			
NPPA	TGATCGATCTGCCCTCCTAA	TCCTCCCTGGCTGTTATCTTC	natriuretic peptide A
**Housekeeping gene**			
18S rRNA	CTCAACACGGGAAACCTCAC	CGCTCCACCAACTAAGAACG	18s ribosomal RNA
