# Supplementary material for: Metabolic Maturation Exaggerates Abnormal Calcium Handling in a Lamp2 Knockout Human Pluripotent Stem Cell-Derived Cardiomyocyte Model of Danon Disease
Source: Biomolecules. 2022 Dec 29;13(1):69. doi: 10.3390/biom13010069 (PMC9855424; doi:10.3390/biom13010069)
Supplement: Supplementary file 1 [file biomolecules-13-00069-s001.zip › Supplemental Table S2.pdf]

Supplemental Table S2. Summary of antibodies used in the current study.

Name	Dilution		Company	Cat. No.
	ICC / IF	WB		
**Primary Antibody**				
Anti-α-Actinin (Sarcomere) Mouse monoclonal	1:800	1:2500	Sigma Aldrich	A-7811
Anti-Cardiac Troponin T Rabbit IgG	1:1000	1:1000	Abcam	Ab-45932
Anti-LAMP-2 (H4B4) Mouse monoclonal IgG		1:500	Santa Cruz	sc-18822
Anti-GAPDH (D411) Mouse monoclonal IgG		1:1000	Santa Cruz	sc-47724
Anti-LC3A/B Rabbit		1:1000	Cell Signaling	4108S
Anti-CAMKII Delta Isoforms (2/4/9/11/11a) Rabbit pAb		1:5000	Badrilla	A010-56AP
Anti-CAMKII (pThr286 or 287) Rabbit pAb		1:1000	Badrilla	A010-50AP
Anti-OCT4A (C30A3) Rabbit mAb	1:400		Cell Signaling	2840S
Anti-SOX2 (L1D6A2) Mouse mAb	1:400		Cell Signaling	4900S
Anti-NANOG (D73G4) XP Rabbit mAb	1:200		Cell Signaling	4903S
Anti-SSEA-4 (MC813) Mouse mAb	1:500		Cell Signaling	4755S
Anti-beta3-Tubulin (D7169) XP Rabbit mAb	1:300		Cell Signaling	5568S
Anti-Brachury (D2Z3J) Rabbit mAb	1:600		Cell Signaling	81694S
Anti-SOX17 (D1T8M) Rabbit mAb	1:3200		Cell Signaling	81778S
**Secondary Antibody**				
AlexaFluor488 Goat anti-Rabbit IgG(H+L)	1:1000		Thermo Scientific	A11008
AlexaFluor594 Goat anti-Rabbit IgG(H+L)	1:1000		Thermo Scientific	A110012
AlexaFluor488 Goat anti-Mouse IgG(H+L)	1:1000		Thermo Scientific	A11001
AlexaFluor594 Goat anti-Mouse IgG(H+L)	1:1000		Thermo Scientific	A11005
AlexaFluor647 Goat anti-Mouse IgG (H+L)	1:1000		Thermo Scientific	A21235
Anti-rabbit IgG HRP-linked Antibody		1:2500	Cell Signaling	7074S
Anti-mouse IgG HRP-linked Antibody		1:10000	Cell Signaling	7076S
