# Supplementary material for: Metabolic Maturation Exaggerates Abnormal Calcium Handling in a Lamp2 Knockout Human Pluripotent Stem Cell-Derived Cardiomyocyte Model of Danon Disease
Source: Biomolecules. 2022 Dec 29;13(1):69. doi: 10.3390/biom13010069 (PMC9855424; doi:10.3390/biom13010069)
Supplement: Supplementary file 1 [file biomolecules-13-00069-s001.zip › Supplemental Table S3.pdf]

Supplemental Table S3. Summary of the sequences of sgRNA off-target primers used in the current study.

Genomic position	Primer sequences		Product size (bp)
	Forward	Reverse	
**sgRNA 1 target**: CATTATATGTCACAGTGCCA TGG			
**Off-target site 1**: chr2: -58244737*: CATTCTATGACACAGTGCCA CAG			
Primer set 1	CTCCCAGTTCCCACCAGTAA	TTTGCGTGATCAAATCCAAA	360
Primer set 2	CTCCCAGTTCCCACCAGTAA	AAACAGGCAAGAGCAAACTGA	402
**Off-target site 2**: chr21: +17964866: TAATATGTGTCACAGTGCCA CAG			
Primer set 1	ATTTGGCAGAGGCTTCAGTG	AAACAGGACATGGGGAAACC	462
Primer set 2	TCCTGGTTTCCTGAGTGTCC	TGGTTTCCAGATGTTCTTGG	304
**Off-target site 3**: chr1: +244973646: CGTTTTATTTCACAGTGCCA CGG			
Primer set 1	TTGTGCAGAGTGCTTGGAAC	TGAGCACAATAGCCCCTTCT	481
Primer set 2	GGTCTCTTTTCCCCTCTGCT	TGAGCACAATAGCCCCTTCT	321
**Off-target site 4**: chr11: +121235399: CAGTTTTTGTCACAGTGCCA TGG			
Primer set 1	TGCTGAAGCTGCAGTGAGTT	GCATTTCCAAAATTGCACCT	378
Primer set 2	GGCGCACACGCTTATATCTT	GCATTTCCAAAATTGCACCT	534
**Off-target site 5**: chr15: +100726923: GATGATATGTCCCAGTGCCA TAG			
Primer set 1	TACCATCCCCATTACCCTGA	ATGAGAACAATGCCCCTGAC	419
Primer set 2	CTATGCTCCCTCCTCACTGC	ATGAGAACAATGCCCCTGAC	469
**sgRNA 2 target:** GCATAAGACCGCACAGCTCC TGG			
**Off-target site 1**: chr2: chr10: -48464924: GAATGAGACCTCACAGCTCC TGG			
Primer set 1	AAGCCTCTTGGGAGGCTAAG	AACAGGTTCAGGGAGGAGGT	514
Primer set 2	AAGCCTCTTGGGAGGCTAAG	GGGAAAGTAGGCGATGTTCA	355
**Off-target site 2**: chr8: +111572: CCAGGAGCTGTCCAGTCT TAT			
Primer set 1	GATCCTAGCCTGCACTGCTC	CTCCATGTCATCTCCCTGCT	324
Primer set 2	GCTGCAGGCTTCAGTCTACC	GCTTCTTTGGGACTCTGCTG	479
**Off-target site 3**: chr6: -170696611: ACATAAGACTGGACAGCTCC TGG			
Primer set 1	GCTTCTTTGGGACTCTGCTG	TAGAAGCTGCTGTGCCTGAA	344
Primer set 2	GCTTCTTTGGGACTCTGCTG	GCTGCAGGCTTCAGTCTACC	479
**Off-target site 4**: chr1: +243006185: ACATAAGACTGGACAGCTCC TGG			
Primer set 1	GCTTCTTTGGGACTCTGCTG	GCTGCAGGCTTCAGTCTACC	462
Primer set 2	CTCCATGTCATCTCCCTGCT	GATCCTAGCCTGCACTGCTC	307
**Off-target site 5**: chr1: +223903393: ACATAAGACTGGACAGCTCC TGG			
Primer set 1	GTTCCTGAGGGACAGAGACG	GGAGGTGACCAATCTTGGAA	392
Primer set 2	GCTTCTTTGGGACTCTGCTG	GCTGCAGGCTTCAGTCTACC	479

*: ”+” and “–“ indicate forward strand and reverse strand
